# Activated 4E-BP1 represses tumourigenesis and IGF-I-mediated activation of the eIF4F complex in mesothelioma

**DOI:** 10.1038/sj.bjc.6605184

**Published:** 2009-07-14

**Authors:** B A Jacobson, A De, M G Kratzke, M R Patel, J Jay-Dixon, B A Whitson, A A Sadiq, P B Bitterman, V A Polunovsky, R A Kratzke

**Affiliations:** 1 Department of Medicine, University of Minnesota, Minneapolis, MN 55455, USA; 2 Research Service, Minneapolis Veterans Affairs Medical Center, Minneapolis, MN, 55417, USA; 3 Department of Pharmacology, University of Minnesota, Minneapolis, MN 55455, USA; 4 Department of Surgery, University of Minnesota, Minneapolis, MN 55455, USA

**Keywords:** 4E-BP1, translation, cap-dependent, eIF4E, eIF4F, IGF-I

## Abstract

**Background:**

Insulin-like growth factor (IGF)-I signalling stimulates proliferation, survival, and invasion in malignant mesothelioma and other tumour types. Studies have found that tumourigenesis is linked to dysregulation of cap-dependent protein translation.

**Methods:**

The effect of IGF stimulation on cap-mediated translation activation in mesothelioma cell lines was studied using binding assays to a synthetic 7-methyl GTP-cap analogue. In addition, cap-mediated translation was genetically repressed in these cells with a dominant active motive of 4E-BP1.

**Results:**

In most mesothelioma cell lines, IGF-I stimulation resulted in a hyperphosphorylation-mediated inactivation of 4E-BP1 compared with that in normal mesothelial cells. An inhibitor of Akt diminished IGF-I-mediated phosphorylation of 4E-BP1, whereas inhibiting MAPK signalling had no such effect. IGF-I stimulation resulted in the activation of the cap-mediated translation complex as indicated by an increased eIF4G/eIF4E ratio in cap-affinity assays. Akt inhibition reversed the eIF4G/eIF4E ratio. Mesothelioma cells transfected with an activated 4E-BP1 protein (4E-BP1^A37/A46^) were resistant to IGF-I-mediated growth, motility, and colony formation. In a murine xenograft model, mesothelioma cells expressing the dominant active 4E-BP1^A37/A46^ repressor protein showed abrogated tumourigenicity compared with control tumours.

**Conclusion:**

IGF-I signalling in mesothelioma cells drives cell proliferation, motility, and tumourigenesis through its ability to activate cap-mediated protein translation complex through PI3K/Akt/mTOR signalling.

Malignant mesothelioma is an aggressive cancer arising from the serosal lining of the pleural or peritoneal cavity. Along with asbestos exposure, simian virus 40 infection has been implicated in the development of mesothelioma. Despite attempts to treat mesothelioma with multimodal therapies, estimates of median survival are usually less than 1 year (Whitson and Kratzke, 2006; Lee *et al*, 2007). In the United States, nearly 3000 deaths are attributed to mesothelioma annually (Britton, 2002). The pathogenesis of mesothelioma involves multiple-signalling pathways, including vascular endothelial growth factor, epidermal growth factor, *wnt,* and insulin-like growth factor-I (IGF-I) (Whitson and Kratzke, 2006; Lee *et al*, 2007). A recent study has also demonstrated that both the Ras/mitogen-activated protein kinase (MAPK) pathway and the phosphatidylinositol 3′ kinase (PI3K)/Akt pathway are activated in mesothelioma cells compared with non-transformed mesothelial cells (Patel *et al*, 2007). Many studies have found that the risk of developing several common cancers is linked to elevated levels of circulating IGF-I, as well as to an increased ratio of IGF-I to inhibitory-binding proteins (reviewed in (Jerome *et al*, 2003)). An array-based study found the presence of altered transcript profiles of genes from the IGF-I axis (Hoang *et al*, 2004a). In a follow-up study, mRNA levels of 12 components of the IGF-I axis were elevated in mesothelioma cells compared with that in a mesothelial cell line (Hoang *et al*, 2004b). Four mRNA levels, in particular, were increased: IGF-I (43.3-fold), insulin receptor substrate (IRS)-2 (24.0-fold), IRS-1 (4.4-fold), and IGF1R (2.6-fold). This suggests that an activated IGF-I axis contributes to the malignant phenotype of mesothelioma. In addition, the activation of IGF1R adaptor proteins (IRS-1 and IRS-2) has been shown to contribute to the proliferative or motility phenotype of mesothelioma cells. These previous studies along with others (Lee *et al*, 1993) demonstrate the importance of the IGF-I system in the pathogenesis of mesothelioma.

In normal cells, IGF-I binds to IGF1R and activates its kinase activity, leading to the targeting of its corresponding adaptor proteins, including IRS-1, IRS-2, and SHC. These substrates initiate phosphorylation cascades, resulting in the activation of the extracellular signal-regulated kinase (ERK) 1/2 and PI3K/Akt pathways and leading to cell proliferation and survival (reviewed in (Sachdev and Yee, 2006), (Haddad and Yee, 2006)). Among the probable downstream targets of these pathways is the cap-mediated translation complex. In IGF-I-driven cancers, it is highly probable that the cancer-specific effects of IGF1R activation are mediated in part through the activation of cap-mediated translation.

Deranged cap-dependent protein translation is implicated in tumourigenesis in multiple tumour types (Jacobson *et al*, 2006), including mesothelioma (Patel *et al*, 2007). The rate-limiting step in the initiation of mRNA translation is the binding of eukaryotic initiation factor 4E (eIF4E) to the 5′-cap structure (m7GpppN) of mRNA. Overexpression of eIF4E is observed in many solid tumours. Under normal cellular conditions, eIF4E is negatively regulated by 4E-BP proteins. 4E-BP1 blocks the interaction between eIF4E and the scaffolding protein eIF4G, inhibiting the formation of the active eIF4F complex and suppressing cap-mediated translation. 4E-BP1 undergoes hierarchical phosphorylation during mitogenic stimulation, which decreases its affinity for eIF4E and results in the activation of translation (De Benedetti and Graff, 2004; Polunovsky and Bitterman, 2006). In cancer, elevated levels of active eIF4F increase the translation of “weak” mRNAs with long structured 5′-untranslated regions, which typically encode anti-apoptotic proteins and growth regulatory factors contributing to tumourigenesis (Graff and Zimmer, 2003; Thumma and Kratzke, 2007).

This study examines the importance of cap-dependent translation in mediating the effects of the IGF-I axis in mesothelioma. Evidence is presented establishing the fact that activation of both PI3K and ERK1/2 pathways after IGF-I stimulation leads to a stimulation of the eIF4F complex. Furthermore, a dominant active 4E-BP1 results in the repression of translation and disrupts IGF-I-mediated motility and proliferation of mesothelioma cells. Expression of the dominant active 4E-BP1 resulted in significantly decreased tumourigenicity of murine xenografts. These results demonstrate that the malignant phenotype and tumourigenicity conferred upon mesothelioma by the IGF-I pathway is dependent upon the activation of cap-dependent translation.

## Materials and methods

### Cell lines and cell culture

Mesothelioma cell lines were obtained from either the American Type Culture Collection or from Frederick Kaye (NCI). The medium used for H513, H2052, H2373, H2461, and H2596 was RPMI (Gibco, Invitrogen, Carlsbad, CA, USA), containing 10% calf serum (Biofluids, Rockville, MD, USA). LP9 cells, a non-transformed human mesothelial cell line, were obtained from the National Institute on Aging Cell Repository at passage five. They were maintained in a medium containing a 1 : 1 ratio of M199 and MCDB10 basal medium (Sigma), supplemented with 15% calf serum, 10 ng ml^−1^ EGF, and 0.4 *μ*g ml^−1^ hydrocortisone.

IGF-I stimulation was carried out on cells grown to 50% confluence. Cells were washed twice with phosphate-buffered saline (PBS) and grown in a serum-free medium (SFM) for 14–16 h. Also added at this time to the appropriate plates was the PI3K inhibitor, LY294002 (Promega, Madison, WI, USA) (20 *μ*M), or the MAPK kinase (MAPKK or MEK) inhibitor, U0126 (Promega) (10 *μ*M). The medium was then replaced with fresh SFM containing or not containing 5 nM IGF-I (Gro Pep) for the indicated times and, where indicated, fresh pathway inhibitors were also added. All cells were maintained at 37°C in 5% CO_2_.

### Cell lysate preparation

Cell lysate was prepared for the cap-affinity assays as follows: Cells were washed with PBS, scraped from the plate in the presence of 1 ml PBS, centrifuged, and resuspended in five times the pellet volume of freeze–thaw lysis buffer (50 mM Tris-HCl pH 7.5, 150 mM NaCl, 50 mM NaF, 1 mM EDTA, 10 mM tetrasodium pyrophosphate), supplemented with a protease inhibitor cocktail (Roche, Basel, Switzerland) and a phosphatase inhibitor cocktail (Sigma). The cells were subjected to three consecutive freeze (15 min at −80°C) and thaw (2 min at 37°C) cycles, followed by centrifugation (14K r.p.m., 4°C) for 10 min. For the purpose of studying membrane-bound proteins, a second batch of cell lysate was prepared from cells processed under identical conditions but in the detergent-containing lysis buffer TNESV (50 mM Tris-HCl, pH 7.4; 1% NP-40; 2 mM EDTA, pH 8.0; 0.1 M NaCl), supplemented with both protease and phosphatase inhibitors. Lysate protein concentrations were determined by Bradford assay and stored at −80°C.

### Immunoblot analysis

Protein samples were separated by SDS-PAGE on either straight 15% or an 8–15% gradient (cap-affinity assay samples) and then transferred to a PVDF membrane (Hybond-P, Amersham Biosciences, Piscataway, NJ, USA). Membranes were blocked in 5% non-fat dry milk for 1 h at room temperature in Tris-buffered saline Tween 20 (TBST: 0.15 M NaCl; 0.01 M Tris-HCl, pH 7.6; 0.05% Tween 20), rinsed, and incubated for 1 h at room temperature with the appropriate primary antibody. Blots were probed separately with either rabbit α-eIF4GI antibody (kindly provided by Nahum Sonenberg, McGill University Montreal, Quebec, Canada) at a 1 : 2500 dilution, rabbit α-IGF1R antibody (Santa Cruz Biotechnology Inc., Santa Cruz, CA, USA) at a 1 : 1000 dilution, rabbit α-phospho-IGF1R antibody (Cell Signaling, Danvers, MA, USA) at a 1 : 1000 dilution, rabbit α-Akt antibody (Cell Signaling) at a 1 : 1000 dilution, rabbit α-phospho-Akt (ser473) antibody (Cell Signaling) at a 1 : 1000 dilution, rabbit α-MAPK antibody (Cell Signaling) at a 1 : 1000 dilution, rabbit α-phospho-MAPK (Thr202/Tyr204) antibody (Cell Signaling) at a 1 : 1000 dilution, rabbit α-4E-BP1 antibody (Abcam Inc., Cambridge, MA, USA) at a 1 : 2500 dilution, α-phospho-4E-BP1 (ser65) antibody (Cell Signaling) at a 1 : 1000 dilution, α-eIF4E antibody (BD Biosciences, San Jose, CA, USA) at a 1 : 500 dilution, mouse α-actin (Sigma) at a 1 : 10 000 dilution, or rat α-HA antibody (Roche) at a 1 : 2000 dilution to detect the haemagglutinin-tagged HA-4E-BP1^A37/A46^ proteins. The blots were washed thrice in TBST before incubation with appropriate horseradish peroxidase-labeled secondary antibodies, followed by incubation for 1 h at room temperature and three more washes in TBST. Bands of interest were visualised with the ECL Plus western blotting system (Amersham Biosciences, Piscataway, NJ, USA). The density of protein bands was determined using ImageJ, a public domain Java image-processing programme.

### Cell transfection

Mesothelioma cell lines were transfected with the plasmid pACTAG-2 encoding the neomycin resistance gene or with pACTAG *neo*/HA-4E-BP1^A37/A46^. The cells were plated at 50% confluence and transfected (Lipofectin reagent (Invitrogen Life Technologies, Carlsbad, CA, USA)) with 5 *μ*g of the appropriate plasmid DNA. After 72 h, the growth medium was replaced with medium containing G-418 (Cellgro, Manassas, VA, USA) (250 *μ*g ml^−1^). Resistant colonies were collected and transferred to a fresh plate and propagated.

### Cloning efficiency assay

Cell lines were transfected according to the procedure as described above, with one exception. When resistant colonies were of appropriate size, plates were fixed in 10% methanol and 5% acetic acid for 10 min, stained in 0.1% crystal violet/20% ethanol for 5 min, and scored for G-418-resistant colonies as previously described (Kratzke *et al*, 1995). All transfections were carried out in triplicate.

### Cell migration assay

Cell motility was assessed by seeding 5 × 10^3^ cells into coated wells of BioCoat Invasion Chambers (BD Biosciences). Twenty-four hours later, the cells were washed twice with PBS and cultured in SFM with or without 5 nM IGF-I. After 24 h of incubation, the cells remaining on the top of the membrane were removed with cotton swabs, whereas cells migrating through the 8 *μ*m-sized pores onto the opposite side of the membrane were fixed (100% methanol), stained (0.1% crystal violet), and then counted microscopically. All experiments were carried out in triplicate.

### Cell proliferation assay

Cells were seeded as triplicate sets into 96-well plates with 6000 cells per well for H2373, H2373 empty vector, and H2373 4E-BP1^A37/A46^, and with 3000 cells per well for H2461, H2461 empty vector, and H2461 4E-BP1^A37/A46^. After 24 h, the cells were switched to SFM and incubated for another 24 h in the presence or absence of 5 nM IGF-I. After incubation, cell growth was measured using the Cell Counting Kit-8 (Dojindo Molecular Technologies, Rockville, MD, USA) according to the manufacturer's protocol. At the appropriate time, 10 *μ*l of reagent was added to each well and incubated at 37°C for 4 h. Absorbance at 450 nm for each well was read on a microplate reader. Each experiment was repeated twice with similar results for each cell line.

### *In vitro* cap-affinity assay

Cap-affinity assay was performed as before (Jacobson *et al*, 2006); briefly, 300 *μ*l (1 *μ*g *μ*l^−1^) of cell lysate was incubated while mixing for 2 h at 4 °C with 50 *μ*l of suspended (50% mixture) 7-methyl GTP-Sepharose4B (Amersham Biosciences) to capture eIF4E and its binding partners, eIF4G and 4E-BP1. The captured proteins were eluted with 35 *μ*l of elution buffer (25 mM Tris-HCl pH 7.5, 150 mM KCl) containing 100 *μ*M of 7-methylguanosine 5′-triphosphate (Sigma-Aldrich, St Louis, MO, USA) and were prepared for immunoblotting.

### Xenograft tumourigenicity assay

H2373 cells (2.1 × 10^7^) stably transfected with pACTAG *neo* or with pACTAG *neo*/HA-4E-BP1^A37/A46^ were suspended in 0.5 ml of PBS and injected subcutaneously into the left or right flank, respectively, of five nude mice (*nu/nu*; Harlan). Tumour formation was quantified after 23 days by weight. Another set of five nude mice were injected with 0.5 ml of PBS containing H2596 cells (2.6 × 10^7^) stably transfected with pACTAG *neo* or pACTAG *neo*/HA-4E-BP1^A37/A46^ into the left or right flank, respectively. Fourteen days after injection, the growth of these tumours was quantified by weight. All animal experiments were carried out under Institutional Animal Care and Use Committee of the Department of Veteran Affairs Medical Center, which conforms with the UKCCCR guidelines (Workman *et al*, 1998).

## Results

### IGF-I-mediated inactivation of 4E-BP1 depends on Akt signalling in mesothelioma

**Figure 1:**
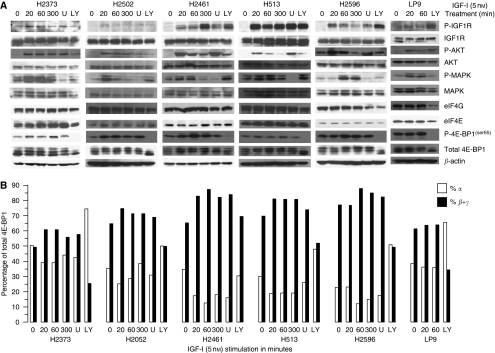
IGF-I stimulation activates downstream-signalling pathways and inactivates 4E-BP1 in mesothelioma cell lines. (**A**) Immunoblot analysis showing phosphorylation and total levels of proteins involved in cell-signalling pathways (IGF1R, Akt, and MAPK) or in initiation of translation (eIF4G, eIF4E, and 4E-BP1) after treatment (in minutes) with and without IGF-I (5 nM) or on treatment with IGF-I (5 nM for 20 min) combined with LY249002 (LY) or U0126 (U). (**B**) Representation of the percentage of 4E-BP1 in hypophosphorylated isoforms (α, open columns) compared with that in hyperphosphorylated isoforms (β + γ, filled columns) for mesothelioma cells and mesothelial control cells (LP9) not treated or treated with IGF-I (5 nM) or IGF-I combined with LY249002 (LY) or U0126 (U) for the indicated times. β-actin represents loading controls for each cell line.

To assess the impact of IGF-I stimulation on the activation of signalling pathways underlying the malignant phenotype, a panel of mesothelioma cell lines and normal mesothelial cells were treated with IGF-I (5 nM) for varying times, followed by immunoblot analysis. Treatment of IGF-I for 20 min stimulated the intrinsic tyrosine kinase activity of IGF1R, resulting in phosphorylation in both mesothelioma cells and control LP9 mesothelial cells (Figure 1A). Increased IGF1R phosphorylation lasted for up to 300 min in two cell lines (H2461, H513), but drifted back to baseline levels in the remaining mesothelioma cells (H2373, H2052, and H2596) and control LP9 mesothelial cells. As expected, total IGF1R levels did not change during the experiment. As shown in earlier studies (Hoang *et al*, 2004b), MAPK and PI3K/Akt were phosphorylated upon IGF-I stimulation for 20 min in all five of the mesothelioma cell lines and control mesothelial cells. Peak levels of MAPK and Akt phosphorylation were reached at 20 min in all mesothelioma cell lines, with the exception of H2596 cells, which peaked at 60 min. Normal LP9 mesothelial cells had a weaker response to IGF-I stimulation. Total Akt and MAPK protein levels remained stable for both the LP9 control cells and mesothelioma cell lines.

Sequential phosphorylation of 4E-BP1 leads to slower electrophoretic migration, with the γ, β, and α isoforms corresponding, respectively, to hyper-, intermediate, and hypophosphorylation. After IGF-I stimulation, slow-migrating γ and β isoforms of 4E-BP1 were phosphorylated in a sustained manner even for up to 300 min in most, but not all, of the mesothelioma cell lines (Figure 1A). The percentage of hypophosphorylated 4E-BP1 decreased when mesothelioma cells were stimulated with IGF-I (Figure 1B). These results reveal a direct correlation between IGF-I stimulation and inactivation of the 4E-BP1 repressor protein.

The roles of Akt and MAPK pathways were investigated next to delineate whether these pathways mediate an IGF-I-induced inactivation of 4E-BP1 in mesothelioma. Akt inhibition with LY294002 strongly diminished the phosphorylation of Akt and its downstream target 4E-BP1 in all five mesothelioma cell lines and control LP9 cells (Figure 1A and B). Although MAPK inhibition with U0126 did suppress the phosphorylation of MAPK, it did not suppress 4E-BP1 phosphorylation as expected (Figure 1A and B). These results confirm that IGF-I-mediated inactivation of 4E-BP1 is dependent upon Akt/mTOR signalling. Finally, the level of eIF4G, the scaffolding component of the eIF4F complex necessary for the initiation of translation, was not affected by IGF-I stimulation and was mildly decreased upon LY294002 treatment in three of the five mesothelioma cell lines (H2052, H513, and H2596) and LP9 cells (Figure 1A). It is known that eIF4G is cleaved by caspase 3 during apoptosis induction. It is possible that treatment with LY249002 decreased eIF4G levels by inducing caspase-mediated cleavage (Prevot *et al*, 2003).

### IGF-I stimulation activates the cap-mediated translation complex in mesothelioma

**Figure 2:**
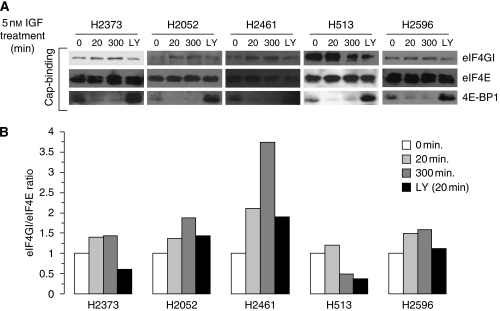
IGF-I stimulation activates cap-mediated translation in mesothelioma. (**A**) After overnight serum starvation, cells were treated or not treated with IGF-I (5 nM) for the indicated times (minutes) or treated with IGF-I combined with LY249002 (LY). Samples were subjected to cap-analogue capture using 7-methyl-GTP-sepharose before immunoblot analysis. (**B**) Depiction of the relative level of eIF4G normalised to eIF4E bound to the cap analogue for each cell line.

On the basis of the findings that IGF-I stimulation promotes phosphorylation of both 4E-BP1 and eIF4E, resulting in the inactive and active forms of these proteins, respectively, it was hypothesised that IGF-I stimulation of mesothelioma cells would result in an enhanced eIF4F function and increased translation. To examine the effects of IGF-I stimulation on cap-dependent translation in mesothelioma, synthetic 7^m^GTP-sepharose beads were used to capture eIF4E and its binding partners, eIF4G and 4E-BP1. Mesothelioma cells were treated with or without IGF-I, and cap-binding assays were performed on cell lysate prepared in the same manner as the assays described in Figure 1. Relatively higher levels of cap-bound eIF4G in cell lysate signify the functional potency of eIF4F, whereas higher levels of cap-captured 4E-BP1 indicate a repression of the eIF4F complex. Consistent with the activation of the cap-mediated translation complex, the quantity of eIF4E-bound eIF4G was increased in most mesothelioma cell lines treated with IGF-I for 20 or 300 min compared with that in untreated cells (Figure 2A, top panel). Furthermore, 4E-BP1 binding decreased substantially (Figure 2A, lowest panel) in most mesothelioma cells treated with IGF-I, indicating an inactivation of 4E-BP1 after phosphorylation. In response to IGF-I stimulation, the ratio of eIF4G to eIF4E was increased 1.5- to 3.5-fold compared with that in untreated cells (Figure 2B). The effect on cap-dependent translation of activating 4E-BP1 by blocking the PI3K/Akt/mTOR-signalling pathway with LY249002 was also examined using the cap-affinity assay. In all mesothelioma cell lines, LY249002 treatment resulted in a decrease in the eIF4G/eIF4E ratio in IGF-I-stimulated cells than in cells treated with only IGF-I (Figure 2B). In some cell lines (H2373, H513, and H2596), the ratio fell to levels at or below that of IGF-I-untreated cells. In addition, the level of eIF4E-bound 4E-BP1 increased in IGF-I stimulated cells treated with LY249002 (Figure 1A), consistent with the suppression of cap-dependent translation.

### Constitutively active 4E-BP1^A37/A46^ inhibits activation of the eIF4F complex in mesothelioma

**Figure 3:**
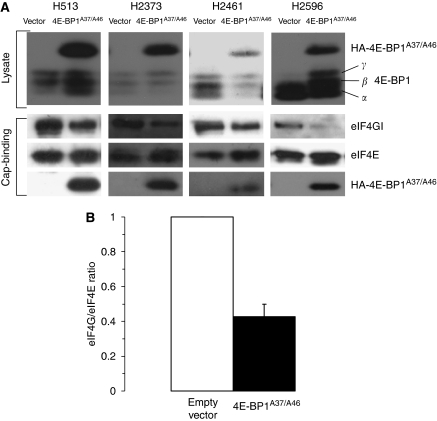
Assembly of cap-dependent initiation complex in mesothelioma is impaired by the production of constitutively active 4E-BP1 (4E-BP1^A37/A46^). (**A**) An assessment of eIF4F integrity utilising a cap-affinity assay of four mesothelioma cell lines ectopically expressing the 4E-BP1^A37/A46^ protein or control. *Top,* steady state levels of exogenous HA-tagged 4E-BP1^A37/A46^ and endogenous 4E-BP1 in cells. *Bottom*, eIF4E and binding partners eIF4G and 4E-BP1^A37/A46^ eluted from 7-methyl-GTP-sepharose resin. (**B**) The average and s.d. for the 4 MM cell lines, relative level of eIF4G normalised to eIF4E bound to the cap analogue comparing cells expressing and not expressing 4E-BP1^A37/A46^.

If cap-mediated translation contributes to the malignant phenotype, then repressing its activity should repress the malignant potential of mesothelioma cells. Mesothelioma cells were selected to ectopically express active 4E-BP1 using a phosphorylation-defective 4E-BP1^A37/A46^. The 4E-BP1^A37/A46^ construct has substituted alanine for threonine at residues 37 and 46, the first two sites of sequential phosphorylation that render 4E-BP1 incapable of phosphorylation and consequently dominantly active (Gingras *et al*, 2001). To assess the ability of 4E-BP1^A37/A46^ to interfere with the assembly of the eIF4F initiation complex, a cap-analogue capture of eIF4E and its binding partners was used, followed by immunoblot analysis. Four mesothelioma cell lines were stably transfected with an empty vector or with the same vector-containing 4E-BP1^A37/A46^. Expression of exogenously produced 4E-BP1^A37/A46^ was present in cell lysate for all four cell lines (Figure 3A, top panel). The HA-tagged ectopically expressed 4E-BP1^A37/A46^ migrates more slowly and is easily monitored (α-HA antibody). For each mesothelioma cell line stably transfected with empty vector, eIF4G avidly bound to eIF4E (Figure 3A, lower panel), which is indicative of a translationally active state. For each cell line, the association of eIF4G to eIF4E was repressed when cells expressed 4E-BP1^A37/A46^ compared with cells transfected with an empty vector (Figure 3A, lower panel). Formation of the eIF4F initiation complex was inhibited in cells expressing 4E-BP1^A37/A46^ by 66.7% (H2373) to 51% (H2461) (Figure 3B). Results from the cap-affinity assay show that the quantity of eIF4E bound to 4E-BP1 inversely correlates with the level of eIF4G bound to eIF4E (Figure 3A). The level of the mutually exclusive cap-bound proteins (eIF4G and 4E-BP1) indicates that ectopically expressed 4E-BP1^A37/A46^ strongly diminishes the activation of the cap-dependent complex.

### Constitutively active 4E-BP1^A37/A46^ inhibits IGF-I-mediated effects on mesothelioma

**Figure 4:**
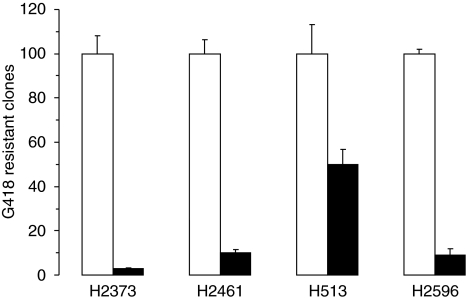
Constitutively active 4E-BP1 hinders mesothelioma colony formation. Mesothelioma cell lines were transfected with empty vector (open columns) or HA-4E-BP1^A37/A46^ (filled columns). G418-resistant clones were counted for each transfection and normalised to the value for empty vector transfection for each cell line.

The previous results demonstrate that IGF-I stimulation of mesothelioma cells directly promotes activation of the eIF4F complex. The next step was to evaluate the link between cap-mediated translation and mesothelioma cell cloning efficiency, proliferation, motility, and tumourigenicity. Each of the four mesothelioma cell lines was transfected with either the dominant active 4E-BP1^A37/A46^ gene or the empty vector. Colonies were counted and normalised to that of the empty vector. In all four mesothelioma cell lines, ectopically expressed 4E-BP1^A37/A46^ exhibited a dramatic impact on cloning efficiency (Figure 4).

**Figure 5:**
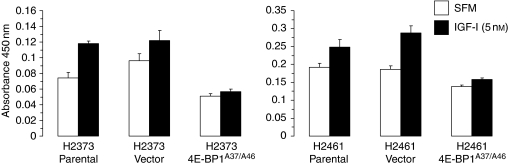
Abrogation of IGF-I-stimulated cell proliferation by a constitutively active 4E-BP1. H2373 and H2461 cell lines expressing dominant active 4E-BP1^A37/A46^ in the presence or absence of IGF-I (5 nM), and 24 h later, cell growth was measured. *Columns,* the mean of three independent determinations of cell number, *bars,* s.d.

**Figure 6:**
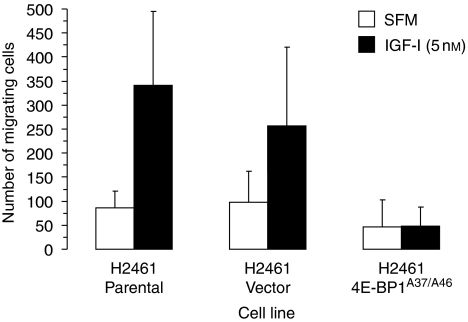
Suppression of IGF-I activated cell motility by disruption of cap-mediated translation. H2461 cells that do and do not produce dominant active 4E-BP1^A37/A46^ in the presence (filled columns) or absence (open columns) of IGF-I (5 nM). After 24 h incubation, cell migration was assayed. *Columns,* the mean of three independent determinations of migrating cells, *bars,* s.d.

To assess the effects on motility and proliferation when cap-mediated translation is repressed, mesothelioma cell lines were stably transfected with empty vector or with the 4E-BP1^A37/A46^ dominant active gene. Transfected cells and control parental cells were grown in SFM in the presence or absence of IGF-I (Figure 5). In both the parental and empty vector-containing cell lines, IGF-I exposure led to increased proliferation compared with that in untreated cells (Figure 5). 4E-BP1^A37/A46^ expression resulted in diminished proliferation. In a similar manner, 4E-BP1^A37/A46^ expression decreased mesothelioma cell motility after IGF-I stimulation (Figure 6), although the results did not reach statistical significance. Parental and empty vector-containing H2461 cells showed a 4- and 2.6-fold enhancement of cell migration, respectively, when exposed to IGF-I than did untreated cells. In contrast, the migration of H2461 cells expressing 4E-BP1^A37/A46^ did not increase in response to IGF-I stimulation. These results demonstrate that the proliferative and migratory responses of mesothelioma cells after IGF-I treatment are blunted by repressing cap-mediated translation.

### Constitutively active 4E-BP1^A37/A46^ abrogates xenograft tumour growth

**Figure 7:**
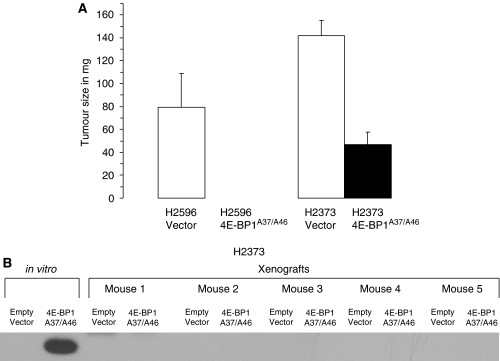
Xenograft tumour growth is abrogated by constitutively active 4E-BP1. (**A**) tumour size for 10 animals (mean±s.e.) from cell lines H2373 and H2596 expressing (filled columns) or not expressing (open columns) 4E-BP1^A37/A46^. *Columns,* the mean of five xenograft measurements of each cell line, *bars,* s.e. (**B**) immunoblot analysis of lysates from mesothelioma cells producing or not producing 4E-BP1^A37/A46^ both before (*in vitro*) and after implantation (xenograft) into the mice. The level of ectopic HA-tagged 4E-BP1 is shown.

The IGF-I stimulation of cap-mediated translation in mesothelioma is ultimately only important if it contributes to the tumourigenic potential of mesothelioma cells. To demonstrate the relationship between cap-mediated translation and *in vivo* tumour formation, H2596 or H2373 cells ectopically expressing 4E-BP1^A37/A46^ or empty vector were injected into nude mice. Animals were killed, tumours were excised, and weighed on days 14 and 27 after implantation of H2596 and H2373 cells, respectively. Expression of 4E-BP1^A37/A46^ caused a marked suppression of tumour growth in these mesothelioma xenografts (Figure 7B). H2373 cells expressing 4E-BP1^A37/A46^ produced tumours that were one-third the size of parental controls. H2596 cells expressing 4E-BP1^A37/A46^ did not produce tumours in any mice. Lysate prepared from H2373 cells expressing empty vector or 4E-BP1^A37/A46^ was subjected to immunoblot analysis both before and after implantation into mice (Figure 7A). The level of expression of the 4E-BP1^A37/A46^ protein diminished during xenograft tumour growth to a nearly undetectable level in H2373 xenograft tumours, suggesting that tumour growth occurred after the selection of cells expressing minimal levels of 4E-BP1^A37/A46^. These results show that suppression of aberrant cap-dependent translation in mesothelioma cells abrogates tumour growth.

## Discussion

Activated cap-mediated protein translation is found in many cancers (Mamane *et al*, 2004). This activation is associated with the preferential translation of a limited number of transcripts responsible for cell proliferation and survival (Graff and Zimmer, 2003; Rajasekhar *et al*, 2003). Enhanced cap-mediated translation in cancer often occurs after either an overexpression of eIF4E, phosphorylation of eIF4E, or an inactivation of the 4E-BP1 repressor protein. In mesothelioma, the primary mechanism for elevated levels of cap-mediated translation seems to be a low-level expression of the 4E-BP1 repressor protein in conjunction with an activation of eIF4E (Patel *et al*, 2007). This is in contrast to previous observations in cancers, such as non-small-cell lung cancer, in which both elevated levels of eIF4E and inactivation of the 4E-BP1 repressor protein are the primary mechanisms for activating the eIF4F complex (Jacobson *et al*, 2006). This study shows that IGF-I stimulation of mesothelioma cells results in a similar hyperphosphorylation and inactivation of 4E-BP1 (Figure 1).

It is well known that IGF-I stimulation of eukaryotic cells can lead to increased protein translation and contribute to the malignant potential in a variety of cell types (Miller and Yee, 2005). In mesothelioma, IGF-I stimulation leads to the downstream activation of both PI3K/Akt and MAPK pathways (Hoang *et al*, 2004b). The observation that IGF-I stimulation is associated with increased phosphorylation of 4E-BP1, allowing for an increase in the binding of the competing eIF4G protein, thus favouring protein translation, is not unexpected and is consistent with IGF-I-driven malignant growth in mesothelioma. In support of the function of the IGF-I axis in mesothelioma development, previous data demonstrate that malignant transformation of mesothelial cells in animal models requires an intact IGF1R molecule (Pass *et al*, 1996). An intact and active IGF-I axis is necessary for both the development and propagation of mesothelioma cells.

Activation of IGF1R is associated with an increase in both mesothelioma cell proliferation and motility (Hoang *et al*, 2004b). In this study, when cap-mediated protein translation was suppressed using the dominantly active mutant protein, 4E-BP1^A37/A46^, mesothelioma cells showed diminished proliferation and motility even under conditions of IGF-I stimulation. IGF-I stimulation of mesothelioma cells also led directly to the activation of the cap-mediated translation complex. The importance of activation or repression of cap-mediated translation in mesothelioma is strongly emphasised by the observation that an enforced repression of translation in mesothelioma cells resulted in the near total loss of tumourigenic potential.

Our finding that cap-mediated protein translation is associated with IGF-I stimulation suggests that IGF1R inhibition is a very attractive therapeutic target in mesothelioma. IGF1R inhibitors are currently in clinical testing and seem to have potent *in vitro* activity against mesothelioma cells (Whitson *et al*, 2006). There are data that these agents are synergistic with cytotoxic chemotherapy, an observation that fits well with the pro-apoptotic effects known to accompany a repression of cap-mediated translation. We have previously shown that an enforced repression of cap-mediated activity can render cancer cells more susceptible to chemotherapeutic-induced apoptosis in non-small-cell lung cancer cells (Jacobson *et al*, 2006). Suppressing IGF1R activation should have the same effect of reducing cap-mediated translation and enhancing chemosensitivity. In addition, the IGF1R-mediated activation of PI3K leads to an acquired resistance to gefitinib, an epidermal growth factor receptor tyrosine kinase inhibitor, in A431 lung cancer cells (Guix *et al*, 2008). The inhibition of both EGFR and IGF1R was required to reverse acquired *in vitro* drug resistance. It is tempting to speculate that both oncogenic pathways converge upon the initiation of translation to mediate their oncogenic effects. If so, the initiation of cap-dependent translation would be an attractive target for new therapies, as it would potentially block the downstream signal of two major oncogenic pathways.

Novel agents are being developed for targeting eIF4E or for disabling the eIF4F molecular complex as a potential cancer therapy (Graff *et al*, 2007, 2008). On the basis of these data demonstrating the important function that IGF-I stimulation and cap-mediated protein translation have in the malignant potential of mesothelioma cells, these therapies could prove to be promising treatments for mesothelioma. In the light of the findings that the effects of IGF1R activation in mesothelioma depend on the activation of the eIF4F complex, it will be interesting to combine IGF1R inhibition with agents designed to target cap-mediated translation.
